# Supplementary material for: The interactome of intact mitochondria by cross-linking mass spectrometry provides evidence for coexisting respiratory supercomplexes
Source: Mol Cell Proteomics. 2017 Dec 8;17(2):216–32. doi: 10.1074/mcp.RA117.000470 (PMC5795388; doi:10.1074/mcp.RA117.000470)
Supplement: Supplemental Data [file supp_RA117.000470_133922_0_supp_23978_fzffwf.zip › spectra_annotation/mito_spectra_annotation/332-1-2-1-7-1.pdf]

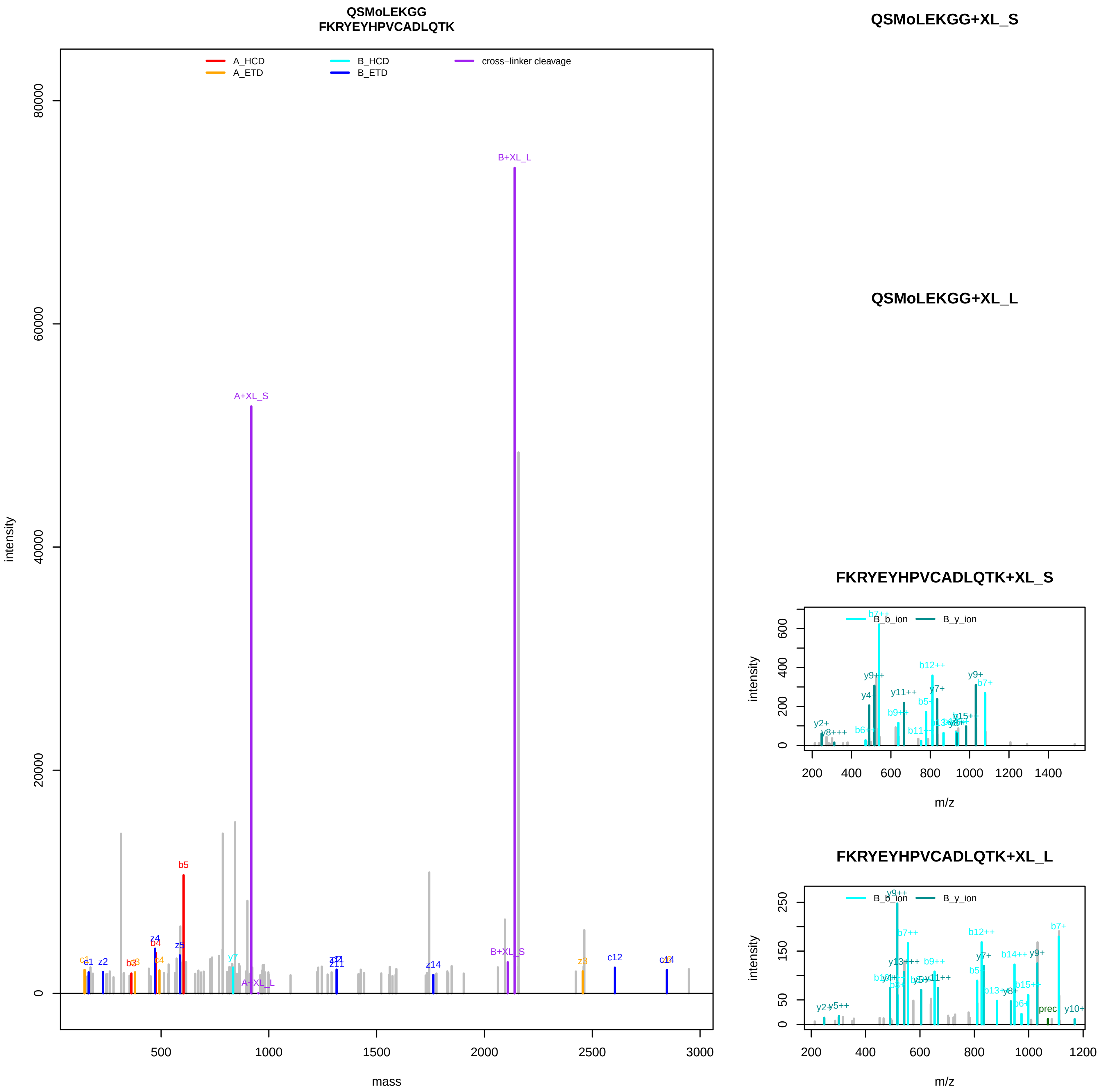
QSMoLEKGG
FKRYEYHPVCADLQTK
A_HCD
A_ETD
B_HCD
B_ETD
cross−linker cleavage
B+XL_L
A+XL_S
A+XL_L
B+XL_S
b5
b4
b3
z4
z5
c1
z2
c4
y7
z14
c12
c14
intensity
mass
QSMoLEKGG+XL_S
QSMoLEKGG+XL_L
FKRYEYHPVCADLQTK+XL_S
FKRYEYHPVCADLQTK+XL_L
B_b_ion
B_y_ion
m/z
intensity
b7++
b12++
y9++
y9+
b7+
y7+
y11++
y4+
b5+
b9++
y15++
b6++
y2+
b11++
y8+++
b14++
b13++
y13+++
y5++
y10+
prec
